# Specific gene expression in type 1 diabetic patients with and without cardiac autonomic neuropathy

**DOI:** 10.1038/s41598-020-62498-7

**Published:** 2020-03-27

**Authors:** Joanna Gastol, Anna Polus, Maria Biela, Urszula Razny, Lukasz Pawlinski, Bogdan Solnica, Beata Kiec-Wilk

**Affiliations:** 1 Department of Metabolic Diseases, University Hospital, Krakow, Poland; 2 Department of Clinical Biochemistry, Jagiellonian University Medical College, Krakow, Poland; 3 Department of Metabolic Diseases, Jagiellonian University Medical College, Krakow, Poland

**Keywords:** Genetics, Molecular medicine

## Abstract

We hypothesized that some molecular pathways might interact to initiate the process of nervous tissue destruction, promoting cardiac autonomic neuropathy (CAN) in the course of diabetes type 1 (T1D). The study group consisted of 60 T1D patients (58.33% women/41.67% men), on standard therapy. The control group consisted of twenty healthy volunteers recruited in accordance with age, gender and body weight. The presence of CAN was documented by the Ewing test method (ProSciCard apparatus). A microarray data analysis was performed using Gene Spring version 13. The microarray results for selected genes were confirmed by real-time PCR (qRT-PCR), using specific TaqMan Gene Expression Assays. Plasma IL‐6 content was measured by an enzyme-linked immunosorbent assay (ELISA). The p < 0.05 value was considered as statistically significant. The microarray analysis, confirmed by qRTPCR, showed significant up-regulation of autophagy, quantity of mitochondria, quality regulatory genes (mTOR, *GABARAPL2*) apoptosis, ER-stress and inflammation (*NFKB1, IL1b, IL1R1, SOD1*), in T1D when compared to the control group. A significantly higher IL-6 protein level was observed in T1D patients, in comparison to the control group. We concluded that the observed changes in gene expression and activation of intracellular pathways give a coherent picture of the important role of oxidative stress in inflammation and the activation of apoptosis in the pathomechanism of DM. The significance of the inflammatory process, confirmed by the increased level of the inflammation biomarker IL-6 in the pathomechanisms of CAN was shown even in patients with properly treated T1D.

## Introduction

The participation of different genes in the pathogenesis of type 1 diabetes T1D has been theoretically postulated and accepted for a long time^1^. However, the genetic risk of T1D has only recently been analysed using genome-wide association studies, identifying over 60 T1D related susceptibility regions within the human genome, marked by single nucleotide polymorphisms^2^. This gene expression analysis revealed changes in genes related to inflammation (cytokines) and endoplasmic reticulum (ER) stress, leading to the activation of apoptosis^3^.

Neuropathy is considered one of the most common major complications of diabetes^4^. It is well established that hyperglycaemia induces oxidative stress in cells such as neurons and immune cells, resulting in the activation of local inflammation and autophagy/apoptosis mechanisms which may be responsible for the pathogenesis and progression of diabetic neuropathy^4,5^. Cardiac autonomic neuropathy (CAN), caused by the destruction of autonomic nerve fibres in the heart and blood vessels, results in abnormalities in heart rate control and vascular dynamics. CAN is a significant cause of morbidity and mortality, associated with a high risk of cardiac arrhythmias and sudden death in DM patients^6^. It is well known that CAN is an important predictor of cardiovascular events in the diabetic population of patients^7^.

Multicentre clinical trials confirmed the stimulation of chronic diabetes complications through the deterioration of metabolic control^8,9^. The non-enzymatic glycation of proteins (AGEs) alter intracellular signalling through proinflammatory cytokines such as IL-6, TNF-α and free radicals, which further propagate diabetic complications^10^. It should be noted however, that the mechanism of this chronic complication is not completely understood. It is believed to partially arise from deficient nerve regeneration due to concomitant inflammatory processes involving the induction of transmembrane glycoprotein 130 (gp130), which is important for IL-6 signal transduction^11^. The induction of gp130 family of cytokines parallel axonal injury, in the experimentally induced diabetic mice, significantly decreased axonal regeneration^12^.

This study’s goal is to follow, by microarray, the possible alteration of blood cell molecular pathways which may be altered to initiate the process of nervous tissue destruction in the course of T1D.

## Material and Methods

Patients were selected from those attending the diabetic clinic at JUMC Department of Metabolic Disorders. The study group consisted of 60 patients with T1D, confirmed with a positive anti-GAD test result. These patients were treated with a standard therapeutic regimen: multiple daily injections (MDI) or continuous subcutaneous insulin infusions (CSII). The patients were randomized into two subgroups depending on the presence or absence of symptoms of autonomic neuropathy.

The control group (n = 20) consisted of healthy non-obese volunteers recruited in accordance to age, gender and body weight. The exclusion criteria for all participants were: age under 20 and above 65 years; metabolic syndrome, cardiovascular diseases, cancer, chronic inflammation; severe liver or kidney failure, iron deficiency anaemia; pregnancy, breast feeding, hormone replacement therapy; anticoagulant treatment; usage of anti-inflammatory drugs and lack of the patient’s written consent to participate in the study. The study protocol was approved by the Jagiellonian University Bioethical Committee and was in accordance with the Declaration of Helsinki. Informed consent was obtained from all individual participants included in the study.

### Evaluation of autonomic neuropathy (CAN)

The presence of CAN was based on the outcome of the reference method using the ProSciCard apparatus (Ewing test)^13^. The severity of the cardiovascular neuropathy in the investigated group was determined by the number of abnormal results (from 3 up to 5) of Ewing test and pathological blood pressure variability [the guidelines for 2018 year of Polish Society of Diabetes]^13^.

Fasting venous blood was sampled from each participant for further tests. Part of the sample was sent for a routine laboratory diagnostic test, and another part was immediately centrifuged for serum isolation, and then transferred into sterile eppendorfs and stored at −20 °C. Whole blood samples were collected for mRNA isolation using PAXgene Blood RNA Tubes (Becton Dickinson) for material stabilization and transport. Each study participant underwent laboratory tests assessing the level of metabolic control, including: HbA1c level, lipidogram, renal markers, liver function tests and TSH levels. Biochemical tests were performed at the main Laboratory Diagnostics Department in the University Hospital, in accordance with standard procedures.

### RNA isolation

Purification of total RNA from human whole blood was performed using the PAXgene Blood RNA Kit, following producer protocol. The RNA quality was analysed using the Tapestation 2200 instrument (Agilent, USA) and quantified by spectrophotometry on the NanoDrop (Thermo Fisher Scientific, Wilmington, DE, USA).

### Microarray

The RNA quality testing resulted in the removal of samples not fulfilling the minimum quality criteria. Amplification of RNA was performed using the Illumina Amplification kit (Ambion, USA). The Quick Amp labelling kit was used for total RNA labelling according to the manufacturer’s protocol. Hybridization of biotin-labelled cRNA to an Illumina chip was performed according to the manufacturer protocol. Arrays were scanned on a HiScan scanner (Illumina, USA).

### Microarrays statistical analysis

**Figure 1:**
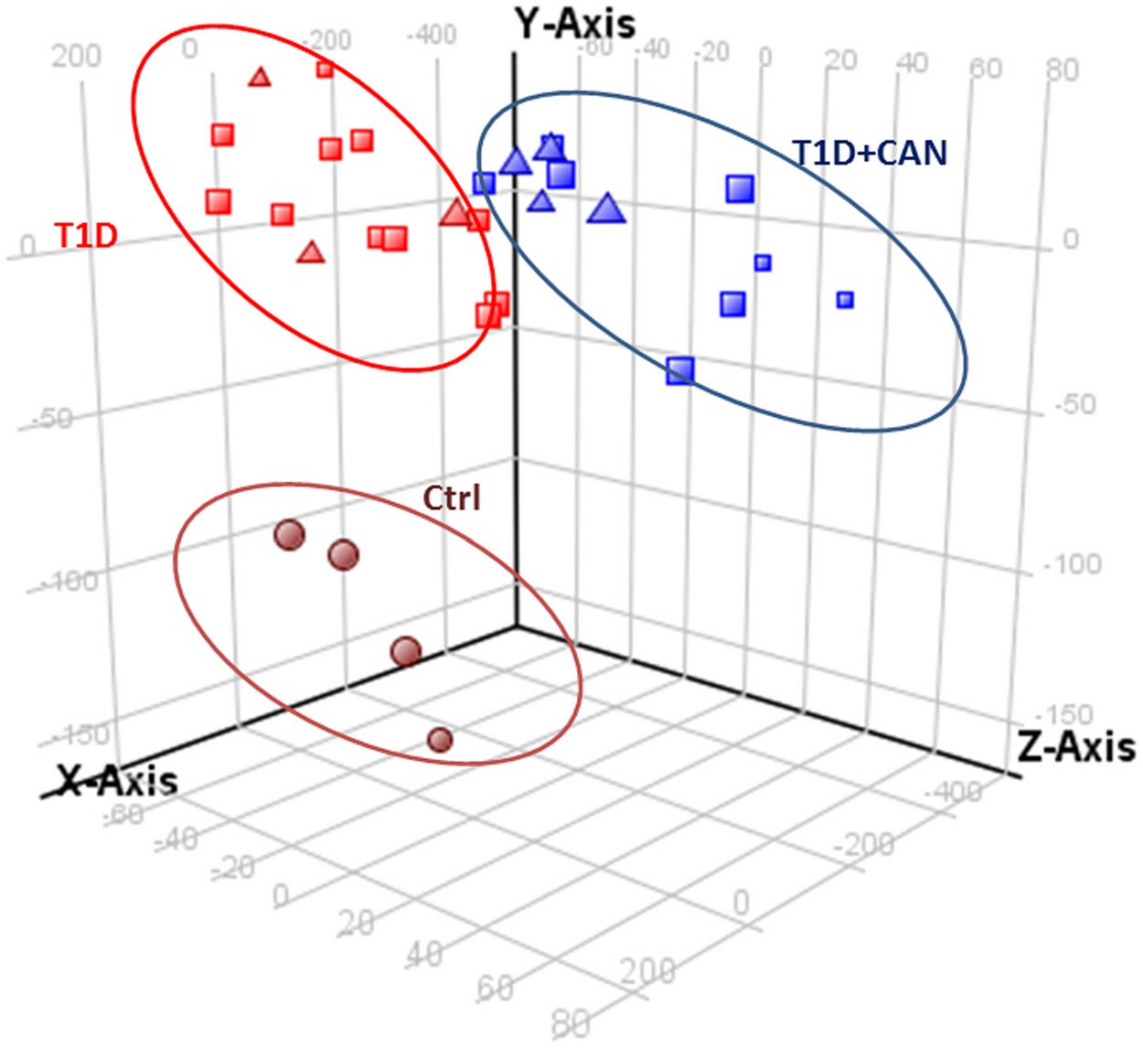
PCA plot analysis of microarray results after the exclusion of outlying test results. The group of diabetic patients without (T1DM) and with cardiac autonomic neuropathy (T1D + CAN), as well as the control group (Ctr).

**Figure 2:**
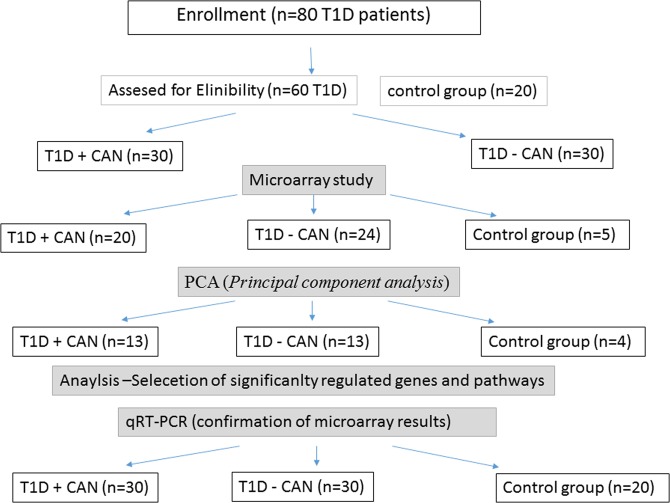
The workflow chart showing the numbers of subjects enrolled in the study; included in the study; and finally included in microarray and qPCR analyses.

**Figure 3:**
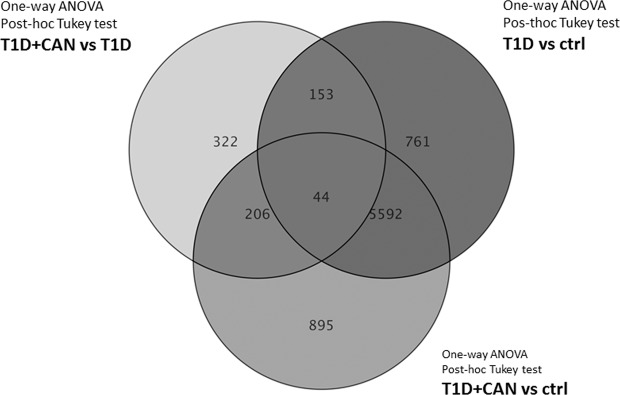
The Venn diagram illustrating the differentially expressed genes identified between each pair of group comparison. The data illustrates significantly regulated genes after one-way ANOVA with post-hoc Tukey test (prepared using GeneSpring tools). The group of diabetic patients without (T1DM) and with cardiac autonomic neuropathy (T1D + CAN), the control group (Ctr).

Microarray was used as a screening method. It was used for selected samples: T1DM without CAN (Ewing test result 1–2), T1DM + CAN group (in Ewing test 4–5 abnormal results). A microarray data analysis was performed using Gene Spring version 13 (Agilent Technologies, Santa Clara, USA). To identify differentially expressed genes, we applied a quartile normalization with background correction to identify the median of all samples for the Illumina chip. Separation of different groups for analysis was carried out by dividing patients into two categories: those with diabetes mellitus without neuropathy (T1D) and those with neuropathy (T1D + CAN); PCA (*Principal component analysis*). PCA was used to discriminate three different groups: T1D, T1D + CAN and ctrl. 3D view of the PCA plot where the three axis are the three principal components (first three by default). Component 1 (X-axis) 57,37%, Component 2 (Y-axis) 4,58%, Component 3 3,47%. Based on the PCA plot analysis, some of the outlier results were excluded from further microarray analysis (Fig. 1), in order to achieve the most homogeneous groups. The outliers on microarray data, in the majority, there were samples of patients that had other factors attributing to the deviations like other chronic complications. From the performed microarray hybridization samples with T1D, only 13 (from 24), T1D + CAN 13 (from 20) and 4 control (from 5) samples were used for further analysis. A smaller number of samples for further analysis were chosen due to the very restrictive clinical characterisation used for patients with the strongest manifestation of CAN (T1D + CAN), in Ewing test 5 abnormal results, and patients without any complications as the T1D group (Fig. 2). The one-way ANOVA with post-hoc Tukey test revealed 6737 significantly regulated genes in T1D + CAN vs. control group, T1D vs. control analysis showed 6550 items; while T1D + CAN vs. T1D covered 725 significantly regulated genes. Among this number, 44 significantly regulated genes, overlapped in all investigated groups. The Venn diagram illustrates the differentially expressed genes identified between each pair of group comparison (Fig. 3). A normalized signal was used for the fold of change (FC) calculation. FC was calculated in relation to the control group (Ctrl), and T1D + CAN vs T1D and used as the expression level. The list of significantly regulated transcripts with p < 0.05 was generated using statistical filtering (one-way ANOVA, as well as Tukey post-hoc test with multiple test correction FDR) (data presented in supplementary materials, Table S1).

In the microarray data analysis, in relation to the biological pathways, the Gene Set Expression Comparison (GSEC) was used for examination of significantly regulated gene sets in pathways (Kegg pathways list) among pre-defined classes of arrays. The list of significantly regulated transcripts with p < 0.05 (FDR) was generated using univariate two-sample T-test (with random variance model). Tests used to find significant gene sets were: LS (least squares)/KS (Kolmogorov-Smirnov) permutation test and Efron-Tibshirani’s GSA max/mean test. The threshold of determining significant gene sets was 0.05.

### Real-time PCR

The microarray results for selected genes were confirmed by real-time PCR. The selection of genes for further analysis by RT-PCR was based on the potentially interesting biological pathways that demonstrated the significantly regulated genes in the Tukey post-hoc test. A real-time PCR analysis was performed on the expanded research group on the basis of the results of the ProSciCard study. 30 CAN candidates with expressed genes (4–5 pathological tests) and 30 T1D of patients with a normal Ewing test result were selected. In addition, 20 healthy people were analysed as a control group (Ctrl). Expression of 18SrRNA was used as a reference gene (Applied Biosystems). One μg of total RNA was reverse transcribed using the reverse transcription kit (High Capacity cDNA Reverse Transcription Kit Thermo Fisher Scientific) with random and oligo dT primers. Subsequently, cDNA was used for real-time PCR. A quantitative real-time polymerase chain reaction (qPCR) was performed using specific TaqMan Gene Expression Assays with a pair of specific PCR primers and a TaqMan probe labelled with FAM (Thermo Fisher Scientific). An amplification was performed using the continuous fluorescence detection 7900 HT Fast Real Time PCR system (Thermo Fisher Scientific). The expression ratio of the target mRNA was normalized to the level of 18 s RNA and compared with the Ctrl and DM + CAN. The data were analysed using the ΔΔCT method and presented as a mean ± standard deviation. Group differences were analysed using a Student T-test. All analyses were performed using Data Assist v3.01. A p < 0.05 value was considered statistically significant.

### IL-6 level estimation

The plasma IL‐6 level was determined using an enzyme-linked immunosorbent assay ELISA (R&D Systems Europe, Ltd, Abingdon, United Kingdom), according to standard protocol. Each sample was performed in duplicates. Within‐ and between‐run imprecision coefficient of variations(CVs) were at level 6% and 7%. The data was presented as mean ± standard deviation. The difference between groups was analysed using a Student T-test. The analysis was performed using Statistica (StatSoft). A p < 0.05 was considered statistically significant. The decision to choose IL-6 protein for further analysis was based on authors’ previous observation, published in *Atherosclerosis* journal^14^. The previous analysis of proteins involved in inflammatory process, in the similar cohort of T1DM patients, gave us arguments to choose IL-6.

### Ethical approval

All procedures performed in this study involving human participants were in accordance with the ethical standards of the Jagiellonian University Bioethical Committee and with the 1964 Helsinki declaration and its later amendments or comparable ethical standards.

## Results

A total of 60 people were qualified for further analysis, including 58.33% women and 41.67% men. The median duration of diabetes was 15 years. Of the examined patients, 58.4% were using MDI, with the rest using CSII. The daily insulin dose (41U vs. 44U) in both groups did not differ significantly. They were examined for the presence of other chronic complications, particularly peripheral neuropathy and retinopathy. None of the patients had advanced painful, peripheral, or severe neuropathy, nor did they demonstrate proliferative retinopathy. The clinical/biochemical characteristics of the analysed groups was presented in the Table 1 (Table 1). The detailed clinical characteristic of investigated group was presented in our previous paper^15^.

**Table 1:** Clinical characteristics of study participants: T1D patients with and without CAN, as well as control group.

PARAMETER (±SD)	T1D (n = 30)	T1D + NSN (n = 30)	control (n = 20)	p
Sex (F/M) %	69/31	60/40	70/30	NA
Age (years)	19.6 ± 5.8	33 ± 17.9	34.4 ± 19.3	NA
Insulin use /day (U)	45 ± 11.4	48 ± 7.2	0	p < 0.0001 (#)
BMI (kg/m^2^)	21.4 ± 2.9	23.1 ± 4.3	23.6 ± 3.7	NA
HbA1c (%)	7.78 ± 1.39	8.62 ± 1.72	5.21 ± 1.72	p < 0.01 (* and #)
C_kr_ < 60 ml/min, n (%)	1 (3.3)	3 (10)	0	p < 0.05 (* and #)

Clinical characteristics of study participants with T1D (n = 60).

C_kr –_Clearance of Creatinine.

*Significant result (p < 0.05) for T1D vs T1D + NSN.

^#^Significant result (p < 0.05) for all patients with T1DM vs control.

Based on the microarray results in the pathway analysis, we focused on genes related to ER-stress, apoptosis, glucose metabolism and inflammation. The genes were categorized according to the Gene Ontology (GO) database using GeneSpring 13 (supplementary materials, Table S1). The entire group of T1D patients demonstrated up-regulation of genes related to ER-stress activation (*ATF6, PRDX6, GCLC, TXNRD1, SOD2*), as well as down-regulation of genes coding proteins involved in glucose transport into the cells (*SLC2A11*) parallel with glycolysis activation, and up-regulation of carnitine production (*SETDB1, DOT1L, SETD2, ALDH9A1*). Additionally, we observed inhibition of alternative pathways that use glucose as a substrate, such as the one involved in the synthesis of glycosaminoglycans (*B3GAT1*). Up-regulation of genes related to proteasome formation was also observed (subunit of proteasome 26S and proteasome 20 complex). Activated ER-stress (confirmed by up-regulation of *ATF6*) provides evidence for the activation of autophagy or apoptosis. These patients additionally demonstrated an up-regulation of the expression of genes related to autophagosome formation (e.g. *ATG3, ULK1, BECN1, DNAJB1, SQSTM1)* but *FRAP1, ATG10, GABARAPL2*, while proteolytic enzymes in the lysosomes (*catepsins*) were down-regulated. Additionally, we discovered down-regulation of the expression of the gene coding the subunit of the protein that builds the H^+^-ATPase lysosome membrane complex, which is designed to activate lysosomal hydrolytic enzymes through lowering the pH of the local environment. The expression of genes associated with apoptosis was also up-regulated (*caspases*, *TNF* family factors and their receptors). We observed an increased gene expression for *NFKB* family factors in T1D; which activates the production of pro-apoptotic proteins from the BCL2 family (supplementary materials, Table S1). We were not able to find significant differences in the gene expression profile between the two groups of T1D patients with and without CAN. Results related to this analysis were presented in Table S1 (supplementary materials, Table S1).

Furthermore, using the microarray results analysis comparing T1D patients with the healthy control, we observed the activation of genes encoding enzymes involved in DNA repair, as well as the inhibition of expression of proteins of complexes I and III, which provide evidence for the impairment of mitochondrial function.

**Figure 4:**
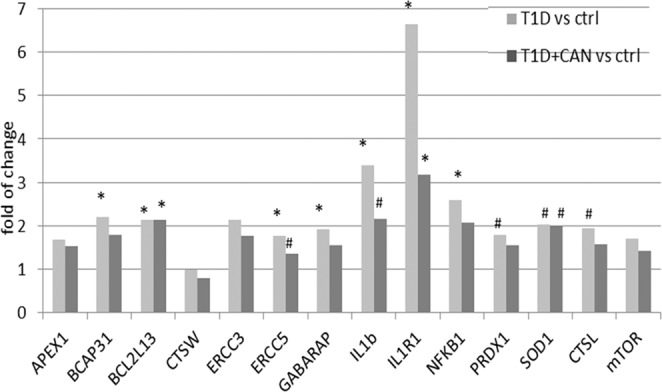
Changes in gene expression of selected genes related to autophagy (*GABARAPL2, FRAP1, CTSL, CTSW*), apoptosis (*BCL2L13*), inflammation (*IL1b, IL1R1, NFKB1*), DNA repair (*APEX1, ERCC3, ERCC5*) and antioxidant enzymes (*PRDX1, SOD1*) in patients with diabetes mellitus. Data are presented as mean ± SD; (*p < 0.05; ^#^p > 0.05 but <0.06−).

The results of microarray analysis showed significantly regulated genes related to autophagy (*GABARAPL2, FRAP1, CTSL, CTSW*), apoptosis (*BCL2L13*), inflammation (*IL1b, IL1R1, NFKB1*), DNA repair (*APEX1, ERCC3, ERCC5*) and antioxidant enzymes (*PRDX1, SOD1*), which was verified and confirmed by qRT-PCR (Fig. 4).

**Figure 5:**
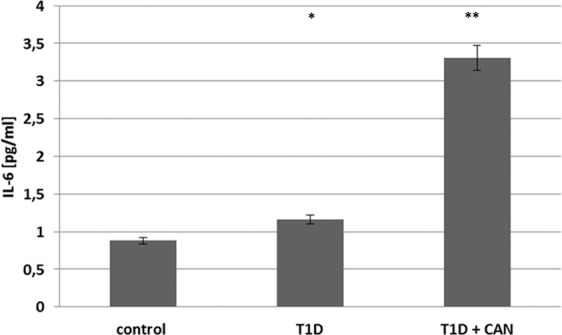
The level of proinflammatory cytokine (IL-6) in the group of diabetic patients without (T1DM) and with cardiac autonomic neuropathy (T1D + CAN), both compared to the control group (Ctr). (*p = 0.0074 T1D + CAN vs T1D; **p = 0.0021 T1D + CAN vs Ctr).

The gene expression analysis demonstrated the activation of pathways associated with the inflammatory process. In order to confirm this observation, the plasma level of IL-6 protein was determined. IL-6 is well-known and reliable inflammatory biomarker, and the choice was dictated by our previous observations in T1DM patients^14^. We observed a significantly higher concentration of this cytokine in the group of T1D + CAN patients, both compared to the group of T1D patients without CAN (p = 0.0074) as well as the control group (p = 0.0021) (Fig. 5).

## Discussion

Despite many years searching for the pathomechanism of the development of chronic T1D complications, including CAN, we still do not have a complete picture. The presented work is one of the first related to this field publications focusing on pathways involved in CAN development in T1D patients, undertaken by analysing blood cell gene expression measured by microarray. In our study, we presented results from a homogeneous T1D group of patients, which minimizes the number of variables interfering with the analysis.

The analysis of gene expression in the investigated group of T1D patients documented the up-regulation of genes related to ER-stress. This observation is in accordance with the *in vitro* study on Schwann cells, where the variation of glucose concentration significantly stimulated ER-stress gene expression in the investigated model^16^. Additionally, the activation of enzyme expression involved in DNA repair, with concomitant inhibition of the expression of components of mitochondrial complexes I and III, pointed to disturbed mitochondrial function, confirming the intensification of oxidative stress^17^. This observation is in accordance with the reported outcome of the experimental animal model study, where mitochondrial dysfunction in cerebellar Purkinje neurons was associated with motor coordination deficits in STZ-diabetic rats, suggesting a novel cellular mechanism of diabetic neuropathy^18^. Various forms of mitochondrial dysfunction such as the inhibition of the respiratory chain, a decrease in ATP generation, and the loss of mitochondrial membrane potential have been previously confirmed in neurodegenerative diseases^19^. Sifuentes-Franco *et al*. documented the role of oxidative stress, mitochondrial dysfunction and impaired autophagy in diabetic polyneuropathy, suggesting that oxidative stress plays a predominant role in neuronal death in DM^20^. Other prospective and clinical analyses of inflammatory biomarkers and distal sensorimotor polyneuropathy pointed to a complex cross-talk between innate and adaptive immunity in the pathogenesis of this disease^21^.

In diabetes, disturbances in blood glucose control cause non-enzymatic protein modification and increased protein degradation by proteasomes and ROS production^5^. In the studied group of patients, we observed increased expression of genes of proteins involved in these events. ER-stress activation through *ATF6* expression points to the activation of autophagy or apoptosis^22,23^. The increased expression of enzymes with antioxidant potential, also provide evidence for the activation of protective mechanisms induced by an abnormal amount of ROS generated within the cells. Activation of gene expression associated with the formation of autophagosomes (*LAMP1, RAB24, GUSB*) is also consistent with this phenomenon^24^. It should be noted however, that patients despite the increased formation of autophagosomes in T1D, the process of removing damaged proteins through autophagy is inefficient due to the inhibition of lysosomal hydrolysis^25^. This observation is in compliance with our results, as we have observed the down-regulation of genes coding lysosomal proteases (*catepsins*). It has been shown that hyperglycaemia in neurons leads to the activation of multiple biochemical and molecular pathways, including protein kinase (PKCs) signalling, which may be crucial for the pathogenesis and progression of diabetic neuropathy^4^.

Additionally, we observed down-regulation of gene expression coding the subunit of protein that builds the H^+^-ATPase lysosome membrane complex, which, activate lysosomal hydrolytic enzymes through lowering the pH of the environment. It may be a potential mechanism of the functional disruption of autophagy, which leads to the activation of cellular apoptosis under the cellular stress in T1D patients^26^. The protective effect of activated autophagy against cellular death in glucose/ROS-mediated apoptosis has been previously recorded^27^. In our study, we observed the up-regulation of the expression of the TNF family and their receptors and the NFKB family, as well as an increase in the expression of caspases genes. These events are documented to be linked with increased biosynthesis of pro-apoptotic proteins from the BCL2 family^28^.

In our T1D patients, we detected a decreased expression of glucose transporters involved in the activation of glycolysis and pathways in which other metabolic substrates could be used as an energy source, such as amino acids and fatty acids. This was evidenced by the increased expression of enzymes participating in carnitine synthesis for the more efficient transport of fatty acids to the mitochondrion. In contrast, we observed inhibition of pathways that use glucose for other purposes, such as the synthesis of glycosaminoglycans. These observations concur with the outcome of another study, where gene expression analysis in pancreatic islets from T1D patients showed not only up-regulation of genes involved in cell death, but significant inhibition of glucose transport as well^3^.

We must stress that we were did not find a significant difference in the measured gene expression profile between sub-groups of T1D patients. The lack of significant differences in gene expression between these groups may be the result of a uniform and intensive model of treatment for these patients, in the form of functional insulin therapy. Good metabolic control, high homogeneity of both T1D groups with and without neuropathy, and relative insulin demand could be reasons for the lack of significant differences in our analysis. On the other hand, despite the optimization of treatment, we found significant differences in genes belonging to various pathways between T1D patients and the healthy control group. This proves that a good clinical control is not successful in suppressing the pathogenic processes leading to the development of diabetes, its progression and the development of chronic complications such as CAN.

The significantly higher gene expression associated with the inflammatory process was expressed in both sub-groups of T1D patients. It should be noted that in T1D + CAN patients demonstrated a significantly higher blood level of IL-6 in comparison to the other analysed groups. This persisted despite intensive insulin therapy with satisfactory metabolic control and strongly argue for the role of inflammatory processes in the development of T1D complications. In the selection of patients for the study, homogeneity of the group was ensured regarding their clinical status and the severity of chronic complications, thus reducing the influence of other factors on the examined biomarkers. The lack of differences in IL-6 gene expression between T1D + CAN and T1D may point to the role of post-translational processes in this mechanism. Another explanation could be that the presence of autonomic neuropathy might result from local, tissue-specific disturbances in metabolic control. It has been shown that both hyper and hypoglycaemic episodes have pro-inflammatory potential in T1D patients^14^. Previous clinical trials have confirmed the association of inflammatory biomarkers with CAN in patients with recently diagnosed T2DM. However, there was no clear association between CAN and IL-6 in this group of patients^29^. A considerable weakness of this study is the limited number of the participants. However, it should be noted that the homogeneity of the groups involving the type of diabetes, race, treatment model, metabolic control and comorbidities helped mitigate many confounding effects. Additionally, the measured changes in gene expression were analysed in the genome of peripheral blood cells. The neuropathy may result from local inflammation related to neuron cell specific regulation of gene expression due to metabolic, ischemic, and/or inflammatory processes. In the absence of clinical input for cardiac interventions and for bioethical reasons, a cardiac conduction system biopsy was not possible in our study model.

Our observations on the changes in gene expression and the activation of intracellular pathways, confirmed by the level of inflammatory proteins, provide a coherent picture of the potential role of aggravated oxidative stress inflammation and the activation of apoptosis in pathomechanisms of CAN in patients with properly treated T1D. We believe that it is crucial to concentrate on the study of specific changes in neuronal tissue in future studies of the pathomechanisms of CAN in type 1 diabetes patients.

## Supplementary information

Table S1.
