# Supplementary material for: Specific gene expression in type 1 diabetic patients with and without cardiac autonomic neuropathy
Source: Sci Rep. 2020 Mar 27;10:5554. doi: 10.1038/s41598-020-62498-7 (PMC7101413; doi:10.1038/s41598-020-62498-7)
Supplement: Supplementary file 1 — Table S1. [file 41598_2020_62498_MOESM1_ESM.docx]

**Specific gene expression in type 1 diabetic patients with and without cardiac autonomic neuropathy.**

Joanna Gastol*^1^, Anna Polus*^2^, Maria Biela^2^, Urszula Razny^2^, Lukasz Pawlinski^1^, Bogdan Solnica^2^, Beata Kiec-Wilk^1,4^.

^1^ Department of Metabolic Diseases, University Hospital, Krakow, Poland

^2^ Department of Clinical Biochemistry, Jagiellonian University Medical College

^3^ Center for Medical Genomics- Omicron, Jagiellonian University Medical College

^4^ Department of Metabolic Diseases, Jagiellonian University Medical College, Krakow, Poland

* these authors equally contributed to the current work.

Corresponding author:

Prof. Beata Kiec-Wilk MD, PhD

Department of Metabolic Diseases Jagiellonian University,

Medical College,

Str. Jakubowskiego 2

30-688 Krakow

Poland

**Table S1.**

The significantly regulated genes in whole blood related to the selected pathways in patients with diabetes mellitus. Significantly regulated transcripts (NCBI symbols) with p < 0.05 (**p** – analysis one-way ANOVA for independent groups, **FDR** - multiple test correction FDR and in comparisons of particular groups, a **bold font** indicates that these results have statistical significance in the Tukey post-hock analysis). (T1D –diabetes mellitus, T1D+CAN-diabetes mellitus with cardiac autonomic neuropathy)

| **NCBI Symbol** | **Accession** | **EntrezID** | **T1D vs ctrl** | **T1D+CAN vs ctrl** | **T1D+CAN vs T1D** | **p (ANOVA)** | **FDR** |
| --- | --- | --- | --- | --- | --- | --- | --- |
| ***Autophagy*** | | | | | | | |
| *autophagosome formation* | | | | | | | |
| ATG10 | NM_031482 | 83734 | -1.64 | **-1.63** | 1.00 | 3.660E-04 | 2.952E-03 |
| ATG3 | NM_022488 | 64422 | **1.35** | **1.27** | -1.06 | 1.800E-06 | 3.551E-05 |
| BECN1 | NM_003766 | 8678 | **1.41** | **1.35** | -1.04 | 9.021E-10 | 4.610E-08 |
| BNIP3L | NM_004331 | 665 | **-1.59** | **-1.81** | -1.14 | 3.026E-03 | 1.580E-02 |
| DNAJB1 | NM_006145 | 3337 | 1.17 | **1.25** | **1.06** | 3.154E-03 | 1.630E-02 |
| GABARAPL1 | NM_031412 | 23710 | **1.28** | 1.18 | -1.09 | 1.352E-02 | 4.991E-02 |
| GABARAPL2 | NM_007285 | 11345 | **-2.12** | **-2.27** | -1.07 | 6.496E-13 | 6.261E-11 |
| GDI1 | NM_001493 | 2664 | **-1.20** | -1.18 | 1.02 | 1.233E-03 | 7.830E-03 |
| HSPA8 | NM_153201 | 3312 | 1.20 | **1.23** | 1.02 | 1.323E-02 | 4.916E-02 |
| LAMP1 | NM_005561 | 3916 | **1.28** | **1.24** | -1.03 | 3.985E-07 | 9.681E-06 |
| LAMP2 | NM_002294 | 3920 | **1.30** | 1.11 | -1.17 | 7.912E-03 | 3.320E-02 |
| RAB24 | NM_001031677 | 53917 | **1.39** | 1.31 | -1.07 | 6.676E-05 | 7.390E-04 |
| RAB7A | NM_004637 | 7879 | **1.27** | 1.19 | -1.07 | 1.912E-03 | 1.100E-02 |
| SQSTM1 | NM_003900 | 8878 | **1.38** | **1.29** | -1.07 | 1.611E-04 | 1.515E-03 |
| ULK1 | NM_003565 | 8408 | **1.35** | 1.21 | -1.11 | 2.514E-04 | 2.176E-03 |
| AP3M2 | NM_006803 | 10947 | **1.24** | **1.28** | 1.03 | 4.161E-04 | 3.273E-03 |
| ATP6V0C | XM_001130742 | 527 | **-1.96** | -1.75 | 1.12 | 1.152E-03 | 7.420E-03 |
| ATP6V1H | NM_213619 | 51606 | **1.27** | **1.24** | -1.02 | 1.426E-08 | 5.276E-07 |
| CD164 | NM_006016 | 8763 | **-1.89** | **-2.28** | -1.21 | 2.133E-07 | 5.673E-06 |
| CTSK | NM_000396 | 1513 | **1.22** | **1.20** | -1.01 | 6.406E-04 | 4.637E-03 |
| CTSO | NM_001334 | 1519 | -1.19 | **-1.26** | -1.06 | 3.087E-04 | 2.570E-03 |
| CTSW | NM_001335 | 1521 | **-1.56** | -1.46 | 1.07 | 1.018E-03 | 6.727E-03 |
| FUCA1 | NM_000147 | 2517 | **1.21** | 1.17 | -1.04 | 2.243E-03 | 1.250E-02 |
| GLA | NM_000169 | 2717 | 1.19 | **1.22** | 1.02 | 1.669E-03 | 9.923E-03 |
| GLB1 | NM_001079811 | 2720 | **1.25** | **1.21** | -1.03 | 1.270E-04 | 1.249E-03 |
| GNPTG | NM_032520 | 84572 | **1.26** | 1.20 | -1.05 | 1.166E-02 | 4.459E-02 |
| GUSB | NM_000181 | 2990 | **1.38** | **1.40** | 1.01 | 4.735E-10 | 2.578E-08 |
| HEXB | NM_000521 | 3074 | **1.22** | 1.15 | -1.06 | 1.010E-02 | 3.998E-02 |
| IDS | NM_006123 | 3423 | **-1.33** | **-1.39** | -1.05 | 7.192E-06 | 1.150E-04 |
| IGF2R | NM_000876 | 3482 | **1.60** | 1.38 | -1.16 | 7.480E-04 | 5.257E-03 |
| LAPTM4A | NM_014713 | 9741 | **-1.26** | **-1.32** | -1.05 | 1.713E-09 | 8.064E-08 |
| LAPTM5 | NM_006762 | 7805 | **1.31** | 1.22 | -1.07 | 7.106E-03 | 3.062E-02 |
| LGMN | NM_001008530 | 5641 | **-1.23** | -1.23 | -1.00 | 3.082E-09 | 1.383E-07 |
| NAGPA | NM_016256 | 51172 | 1.30 | **1.37** | 1.05 | 8.791E-05 | 9.251E-04 |
| PSAP | NM_002778 | 5660 | **1.34** | **1.25** | -1.07 | 8.849E-06 | 1.370E-04 |
| SLC11A1 | NM_000578 | 6556 | **1.71** | **1.73** | 1.01 | 2.417E-04 | 2.107E-03 |
| TCIRG1 | NM_006053 | 10312 | **1.37** | **1.34** | -1.02 | 2.626E-03 | 1.416E-02 |
| TPP1 | NM_000391 | 1200 | **1.38** | **1.29** | -1.07 | 3.801E-04 | 3.041E-03 |
| VTI1B | NM_006370 | 10490 | **-1.55** | **-1.37** | 1.13 | 5.863E-07 | 1.357E-05 |
| *mTOR pathway* | | | | | | | |
| FRAP1 | NM_004958 | 2475 | **1.23** | **1.21** | -1.01 | 2.300E-06 | 4.397E-05 |
| ***apoptosis*** | | | | | | | |
| AKT1 | NM_001014432 | 207 | **1.23** | 1.19 | -1.04 | 2.566E-03 | 1.390E-02 |
| BCL2L1 | NM_138578 | 598 | **-1.25** | -1.15 | 1.09 | 7.600E-04 | 5.327E-03 |
| BCL2L13 | NM_015367 | 23786 | **1.29** | **1.29** | -1.01 | 2.324E-05 | 3.078E-04 |
| BIRC3 | NM_001165 | 330 | 1.38 | **1.40** | 1.02 | 9.053E-03 | 3.683E-02 |
| BNIP3L | NM_004331 | 665 | **-1.59** | **-1.81** | -1.14 | 3.026E-03 | 1.580E-02 |
| BOK | NM_032515 | 666 | **-1.55** | **-1.59** | -1.03 | 9.441E-09 | 3.689E-07 |
| CASP1 | NM_033294 | 834 | **1.43** | **1.40** | -1.02 | 1.797E-05 | 2.486E-04 |
| CASP2 | NM_032982 | 835 | **1.18** | **1.20** | 1.01 | 5.447E-05 | 6.237E-04 |
| IKBKB | NM_001556 | 3551 | **1.19** | **1.19** | -1.00 | 6.669E-05 | 7.386E-04 |
| IKBKG | NM_001099856 | 8517 | **1.35** | **1.36** | 1.01 | 6.495E-10 | 3.433E-08 |
| IRF2 | NM_002199 | 3660 | **-1.64** | **-1.78** | -1.09 | 4.484E-08 | 1.438E-06 |
| MCL1 | NM_021960 | 4170 | **1.38** | 1.18 | -1.17 | 1.213E-03 | 7.727E-03 |
| NFKB1 | NM_003998 | 4790 | **1.27** | **1.25** | -1.01 | 1.503E-05 | 2.138E-04 |
| NFKB2 | NM_001077493 | 4791 | **1.25** | **1.26** | 1.00 | 6.757E-06 | 1.092E-04 |
| NOD2 | NM_022162 | 64127 | **1.50** | **1.40** | -1.07 | 7.389E-04 | 5.205E-03 |
| RIPK1 | NM_003804 | 8737 | **1.38** | **1.33** | -1.04 | 9.446E-09 | 3.689E-07 |
| TNFRSF1A | NM_001065 | 7132 | **1.41** | **1.34** | -1.05 | 3.982E-04 | 3.162E-03 |
| TNFSF10 | NM_003810 | 8743 | **1.38** | 1.31 | -1.05 | 1.004E-02 | 3.977E-02 |
| ***ER-stress*** | | | | | | | |
| ATF6 | NM_007348 | 22926 | **1.33** | **1.26** | -1.05 | 1.445E-03 | 8.859E-03 |
| RXRA | NM_002957 | 6256 | **1.29** | 1.21 | -1.06 | 1.085E-02 | 4.220E-02 |
| PRDX6 | NM_004905 | 9588 | **1.20** | 1.22 | 1.02 | 2.714E-04 | 2.317E-03 |
| GCLC | NM_001498 | 2729 | **-1.25** | **-1.29** | -1.03 | 4.681E-10 | 2.555E-08 |
| TXNRD1 | NM_001093771 | 7296 | **1.32** | **1.17** | -1.12 | 7.958E-06 | 1.255E-04 |
| SOD2 | NM_001024466 | 6648 | **1.60** | **1.46** | -1.10 | 4.980E-03 | 2.322E-02 |
| ***proteasomal degradation*** | | | | | | | |
| PSMA1 | NM_148976 | 5682 | **1.23** | **1.24** | 1.01 | 1.367E-04 | 1.324E-03 |
| PSMA5 | NM_002790 | 5686 | **1.16** | **1.22** | 1.05 | 7.533E-03 | 3.207E-02 |
| PSMB10 | NM_002801 | 5699 | **1.23** | **1.23** | -1.00 | 1.021E-02 | 4.030E-02 |
| PSMB3 | NM_002795 | 5691 | **1.23** | **1.24** | 1.01 | 8.817E-03 | 3.611E-02 |
| PSMB4 | NM_002796 | 5692 | **1.38** | 1.39 | 1.00 | 8.178E-06 | 1.283E-04 |
| PSMB5 | NM_002797 | 5693 | **1.22** | 1.26 | 1.03 | 1.114E-03 | 7.231E-03 |
| PSMB7 | NM_002799 | 5695 | 1.23 | **1.26** | 1.02 | 3.583E-03 | 1.800E-02 |
| PSMB8 | NM_148919 | 5696 | **1.18** | **1.15** | -1.03 | 9.677E-03 | 3.868E-02 |
| PSMB9 | NM_002800 | 5698 | **1.57** | **1.64** | 1.04 | 6.773E-05 | 7.482E-04 |
| PSMC1 | NM_002802 | 5700 | **1.26** | **1.25** | -1.01 | 1.138E-05 | 1.690E-04 |
| PSMC2 | NM_002803 | 5701 | **1.22** | 1.28 | 1.05 | 1.861E-03 | 1.077E-02 |
| PSMC3 | NM_002804 | 5702 | **1.22** | 1.33 | 1.09 | 7.335E-04 | 5.178E-03 |
| PSMC4 | NM_006503 | 5704 | **1.14** | 1.20 | 1.05 | 1.870E-04 | 1.707E-03 |
| PSMC6 | NM_002806 | 5706 | **-1.37** | **-1.47** | -1.07 | 7.125E-13 | 6.785E-11 |
| PSMD1 | NM_002807 | 5707 | 1.21 | **1.19** | -1.02 | 3.871E-04 | 3.090E-03 |
| PSMD2 | NM_002808 | 5708 | **1.39** | **1.35** | -1.03 | 7.504E-07 | 1.683E-05 |
| PSMD6 | NM_014814 | 9861 | **1.23** | **1.24** | 1.01 | 2.167E-03 | 1.216E-02 |
| UBB | NM_018955 | 7314 | **-1.56** | **-1.29** | 1.21 | 4.435E-03 | 2.118E-02 |
| UBE2D1 | NM_003338 | 7321 | **-1.26** | **-1.29** | -1.02 | 3.818E-09 | 1.675E-07 |
| *glucose transport* | | | | | | | |
| SLC2A11 | NM_001024938 | 66035 | **-1.38** | **-1.42** | -1.03 | 8.746E-03 | 3.589E-02 |
| *Zn transport* | | | | | | | |
| SLC39A8 | NM_022154 | 64116 | **-1.27** | **-1.27** | -1.00 | 2.675E-04 | 2.288E-03 |
| *glycolysis* | | | | | | | |
| ACSS2 | NM_001076552 | 55902 | **1.32** | **1.28** | -1.03 | 1.457E-06 | 2.979E-05 |
| ADH4 | NM_000670 | 127 | **-1.43** | **-1.46** | -1.02 | 2.641E-07 | 6.781E-06 |
| ADPGK | NM_031284 | 83440 | **1.22** | 1.12 | -1.09 | 1.247E-04 | 1.230E-03 |
| AKR1A1 | NM_006066 | 10327 | 1.23 | **1.30** | **1.06** | 7.247E-04 | 5.127E-03 |
| ENO1 | NM_001428 | 2023 | 1.29 | **1.36** | **1.05** | 7.639E-04 | 5.348E-03 |
| ENO2 | NM_001975 | 2026 | 1.28 | **1.33** | 1.04 | 2.474E-06 | 4.673E-05 |
| HK1 | NM_033500 | 3098 | **1.29** | 1.27 | -1.02 | 5.938E-04 | 4.359E-03 |
| LDHAL6A | NM_144972 | 160287 | **-1.21** | -1.18 | 1.02 | 3.881E-11 | 2.635E-09 |
| PFKP | NM_002627 | 5214 | 1.13 | **1.29** | 1.14 | 9.449E-03 | 3.802E-02 |
| PGAM4 | NM_001029891 | 441531 | **-4.67** | **-4.87** | -1.04 | 2.589E-29 | 4.376E-26 |
| PGM1 | NM_002633 | 5236 | **1.21** | 1.19 | -1.01 | 3.868E-03 | 1.910E-02 |
| PHKA2 | NM_000292 | 5256 | 1.19 | **1.26** | 1.06 | 1.123E-02 | 4.334E-02 |
| PKM2 | NM_182470 | 5315 | **1.31** | **1.37** | 1.05 | 2.344E-04 | 2.057E-03 |
| ***other energy source*** | | | | | | | |
| *carnitine synthesis* | | | | | | | |
| SETDB1 | NM_012432 | 9869 | **1.33** | **1.32** | -1.00 | 2.716E-08 | 9.260E-07 |
| DOT1L | NM_032482 | 84444 | **1.18** | **1.21** | 1.02 | 4.990E-09 | 2.122E-07 |
| SUV420H1 | NM_017635 | 51111 | **1.17** | 1.12 | -1.05 | 1.064E-03 | 6.973E-03 |
| SETD2 | NM_014159 | 29072 | **1.65** | **1.55** | -1.06 | 1.092E-10 | 6.800E-09 |
| MLL5 | NM_018682 | 55904 | **1.31** | 1.23 | -1.07 | 3.389E-04 | 2.770E-03 |
| ALDH9A1 | NM_000696 | 223 | **1.28** | **1.17** | -1.10 | 5.527E-04 | 4.121E-03 |
| *TCA* | | | | | | | |
| ACLY | NM_198830 | 47 | **1.27** | **1.22** | -1.04 | 2.795E-04 | 2.371E-03 |
| IDH3B | NM_174856 | 3420 | 1.18 | **1.24** | 1.05 | 1.131E-03 | 7.319E-03 |
| SDHA | NM_004168 | 6389 | 1.15 | **1.17** | 1.01 | 7.567E-03 | 3.218E-02 |
| *aminoacids metabolism* | | | | | | | |
| ACAA1 | NM_001607 | 30 | **1.24** | 1.19 | -1.04 | 6.316E-03 | 2.795E-02 |
| EPRS | NM_004446 | 2058 | **1.31** | **1.35** | 1.04 | 4.421E-05 | 5.264E-04 |
| IARS | NM_002161 | 3376 | **1.22** | 1.20 | -1.02 | 2.102E-03 | 1.187E-02 |
| OAT | NM_000274 | 4942 | **1.27** | **1.19** | -1.07 | 2.056E-06 | 4.010E-05 |
| RARS | NM_002887 | 5917 | **1.18** | **1.21** | 1.03 | 7.819E-04 | 5.450E-03 |
| VARS | NM_006295 | 7407 | **1.26** | **1.27** | 1.02 | 2.872E-04 | 2.424E-03 |
| *glutathion metabolism* | | | | | | | |
| GCLM | NM_002061 | 2730 | **-1.69** | **-1.75** | -1.04 | 1.637E-03 | 9.785E-03 |
| *glycosaminoglycans synthesis* | | | | | | | |
| B3GAT1 | NM_054025 | 27087 | **-1.96** | **-1.94** | 1.01 | 6.712E-23 | 2.715E-20 |
| *NAD metabolism* | | | | | | | |
| NAMPT | NM_005746 | 10135 | **1.75** | 1.47 | -1.19 | 2.781E-03 | 1.482E-02 |
| NADSYN1 | NM_018161 | 55191 | **1.24** | 1.22 | -1.02 | 1.809E-04 | 1.664E-03 |
| ***electron transport*** | | | | | | | |
| *I complex* | | | | | | | |
| NDUFA7 | NM_005001 | 4701 | 1.20 | **1.21** | **1.01** | 5.112E-04 | 3.871E-03 |
| NDUFA9 | NM_005002 | 4704 | 1.12 | **1.21** | **1.08** | 1.072E-02 | 4.181E-02 |
| NDUFAB1 | NM_005003 | 4706 | **-1.28** | -1.21 | 1.07 | 3.907E-03 | 1.925E-02 |
| NDUFB8 | NM_005004 | 4714 | **1.20** | 1.27 | 1.06 | 4.679E-03 | 2.211E-02 |
| NDUFC2 | NM_004549 | 4718 | -1.17 | **-1.20** | -1.02 | 5.679E-10 | 3.047E-08 |
| *III complex* | | | | | | | |
| UCRC | NM_013387 | 29796 | **-1.62** | **-1.63** | -1.01 | 1.159E-08 | 4.407E-07 |
| UQCRC2 | NM_003366 | 7385 | **-1.20** | **-1.21** | -1.01 | 7.280E-04 | 5.145E-03 |
| UQCRH | NM_006004 | 7388 | **-3.13** | **-3.10** | 1.01 | 3.392E-15 | 4.680E-13 |
| *IV complex* | | | | | | | |
| SURF1 | NM_003172 | 6834 | **1.20** | **1.22** | 1.01 | 3.152E-04 | 2.614E-03 |
| *ATP synthetase* | | | | | | | |
| ATP5A1 | NM_004046 | 498 | 1.22 | **1.24** | 1.02 | 3.007E-03 | 1.573E-02 |
| ATPIF1 | NM_178191 | 93974 | **1.21** | **1.21** | -1.01 | 9.609E-04 | 6.433E-03 |
| ***adenine nucleotide transport to mitochondrium*** | | | | | | | |
| SLC25A6 | NM_001636 | 293 | **-1.27** | -1.25 | 1.02 | 1.218E-02 | 4.615E-02 |
| ***lipid metabolism*** | | | | | | | |
| LDLR | NM_000527 | 3949 | 1.14 | **1.23** | 1.08 | 2.455E-03 | 1.343E-02 |
| HMGCR | NM_000859 | 3156 | **1.23** | 1.04 | -1.17 | 1.059E-02 | 4.143E-02 |
| ADIPOQ | NM_004797 | 9370 | **1.41** | **1.51** | 1.07 | 1.644E-06 | 3.288E-05 |
| ADIPOR2 | NM_024551 | 79602 | **1.44** | **1.45** | 1.00 | 5.060E-10 | 2.749E-08 |
| DGAT1 | NM_012079 | 8694 | **1.25** | **1.23** | -1.02 | 9.408E-05 | 9.777E-04 |
| LEP | NM_000230 | 3952 | **-1.96** | **-1.97** | -1.00 | 2.835E-03 | 1.505E-02 |
| PLIN2 | NM_001122 | 123 | **1.24** | **1.24** | 1.01 | 5.252E-06 | 8.863E-05 |
| ***inflammatory response*** | | | | | | | |
| CCL4 | NM_002984 | 6351 | **-1.20** | **-1.20** | 1.01 | 1.068E-13 | 1.178E-11 |
| CCL5 | NM_002985 | 6352 | **-1.81** | -1.37 | 1.32 | 3.120E-04 | 2.592E-03 |
| CD4 | NM_000616 | 920 | **-1.36** | -1.32 | 1.03 | 8.429E-03 | 3.491E-02 |
| CXCR4 | NM_001008540 | 7852 | **1.35** | **1.21** | -1.12 | 3.466E-04 | 2.819E-03 |
| CXCR5 | NM_032966 | 643 | **1.41** | 1.46 | 1.04 | 1.320E-02 | 4.904E-02 |
| IL10 | NM_000572 | 3586 | -1.48 | **-1.41** | 1.05 | 3.340E-03 | 1.704E-02 |
| IL1b | NM_000576 | 3553 | **1.50** | **1.44** | -1.04 | 1.115E-03 | 7.233E-03 |
| IL1R | NM_000877 | 3554 | **1.25** | 1.04 | -1.19 | 2.271E-03 | 2.092E-02 |
| NFKB1 | NM_003998 | 4790 | **1.27** | **1.25** | -1.01 | 1.503E-05 | 2.138E-04 |
| NFKB2 | NM_001077493 | 4791 | **1.25** | **1.26** | 1.00 | 6.757E-06 | 1.092E-04 |
| PECAM1 | NM_000442 | 5175 | **1.36** | 1.24 | -1.10 | 2.415E-03 | 1.325E-02 |
| STAT1 | NM_007315 | 6772 | **1.55** | **1.45** | -1.07 | 4.922E-03 | 2.302E-02 |
| STAT2 | NM_005419 | 6773 | **1.52** | **1.43** | -1.06 | 2.930E-03 | 1.542E-02 |
| STAT5A | NM_003152 | 6776 | 1.20 | **1.24** | 1.04 | 3.503E-03 | 1.771E-02 |
| TLR1 | NM_003263 | 7096 | -1.19 | **-1.43** | -1.21 | 6.970E-03 | 3.017E-02 |
| TLR6 | NM_006068 | 10333 | **1.29** | 1.11 | -1.16 | 1.015E-02 | 4.011E-02 |
| TLR8 | NM_016610 | 51311 | **1.36** | 1.23 | -1.11 | 9.189E-03 | 3.726E-02 |
| ***DNA repair*** | | | | | | | |
| *BER* | | | | | | | |
| PARP1 | NM_001618 | 142 | **1.21** | **1.28** | 1.05 | 4.418E-03 | 2.112E-02 |
| PARP3 | NM_001003931 | 10039 | 1.19 | **1.26** | 1.06 | 5.839E-05 | 6.616E-04 |
| PARP4 | NM_006437 | 143 | **1.41** | 1.28 | -1.10 | 5.922E-05 | 6.689E-04 |
| *MMR* | | | | | | | |
| MLH1 | NM_000249 | 4292 | **1.23** | **1.20** | -1.03 | 2.006E-05 | 2.716E-04 |
| RFC1 | NM_002913 | 5981 | **1.19** | **1.17** | -1.02 | 5.138E-05 | 5.936E-04 |
| RPA1 | NM_002945 | 6117 | 1.23 | **1.25** | 1.02 | 2.224E-03 | 1.242E-02 |
| *NER* | | | | | | | |
| DDB1 | NM_001923 | 1642 | 1.25 | **1.30** | 1.04 | 4.394E-04 | 3.422E-03 |
| ERCC3 | NM_000122 | 2071 | **1.39** | **1.39** | 1.01 | 7.906E-09 | 3.168E-07 |
| ERCC5 | NM_000123 | 2073 | **1.43** | **1.36** | -1.05 | 0.000511 | 4.28E-05 |
| XPC | NM_004628 | 7508 | **1.27** | 1.23 | -1.03 | 4.348E-04 | 3.392E-03 |
